# Optimal design of transmitarray antennas via low-cost surrogate modelling

**DOI:** 10.1038/s41598-023-42134-w

**Published:** 2023-09-12

**Authors:** Mehmet A. Belen, Alper Caliskan, Slawomir Koziel, Anna Pietrenko-Dabrowska, Peyman Mahouti

**Affiliations:** 1 Department of Electric and Electronic Engineering, Iskenderun Technical University , 31330 Hatay, Turkey; 2 Department of Electronic and Communication Engineering, Yildiz Technical University, 34220 İstanbul, Turkey; 3 Engineering Optimization & Modeling Center, Department of Technology, Reykjavik University, Menntavegur 1, 102 Reykjavik, Iceland; 4 Faculty of Electronics, Telecommunications and Informatics, Gdansk University of Technology, Narutowicza 11/12, 80-233 Gdansk, Poland

**Keywords:** Computational science, Electrical and electronic engineering

## Abstract

Over the recent years, reflectarrays and transmitarrays have been drawing a considerable attention due to their attractive features, including a possibility of realizing high gain and pencil-like radiation patterns without the employment of complex feeding networks. Among the two, transmitarrays seem to be superior over reflectarrays in terms of achieving high radiation efficiency without the feed blockage. Notwithstanding, the design process of transmitarrays is more intricate due to the necessity of manipulating both the transmission phase and magnitude of its unit elements. For reliability, the design process has to be conducted at the level of full-wave electromagnetic models, which makes direct optimization prohibitive. The most widely used workaround is to employ surrogate modeling techniques to construct fast representations of the unit elements, yet the initial model setup cost is typically high and includes acquisition of thousands of training data points. In this paper, we propose a novel approach to cost-efficient design of transmitarrays. It is based on artificial-intelligence-enabled data-driven surrogates, which can be constructed using only a few hundreds of training data samples, while exhibiting the predictive power sufficient for reliable design. Our methodology is demonstrated by re-using the presented surrogate for the design of high-performance transmitarrays operating at various frequency ranges of 8–14 GHz, 22–28 GHz, and 28–36 GHz.

## Introduction

Antennas belong to the key components of any wireless communications system. Antenna designs can be classified into low (< 10 dBi), middle (10–20 dBi), and high gain structures (> 20 dBi)^1^. Usually, high-gain antennas can be developed using either of the following two methods: (i) based on the optics theory, which manipulates the curvature of design in order to focus the incoming EM waves and form a beam (e.g., parabolic reflectors, lens antennas^2^); (ii) antenna array theory, which is based on manipulation of either geometrical, or feeding properties of each element (e.g., waveguide-slot or microstrip patch antenna arrays^3^). A transmitarray is an alternative high-gain antenna concept, which has been recently attracting a growing interest of researchers^1–3^. Its unique feature is to combine the advantages of both optic and antenna array techniques to develop high-gain structures with low profile and conformal geometries for wide range of applications.

**Figure 1:**
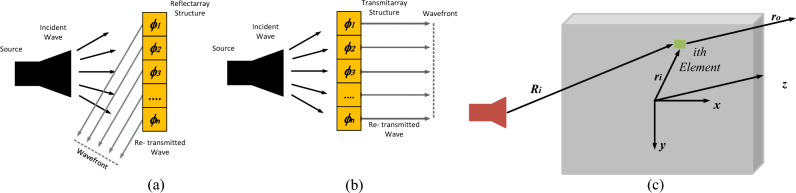
Conceptual illustration of (**a**) reflectarray antenna, (**b**) transmitarray antenna, (**c**) illustration of the phase compensation in transmitarray antenna.

**Figure 2:**
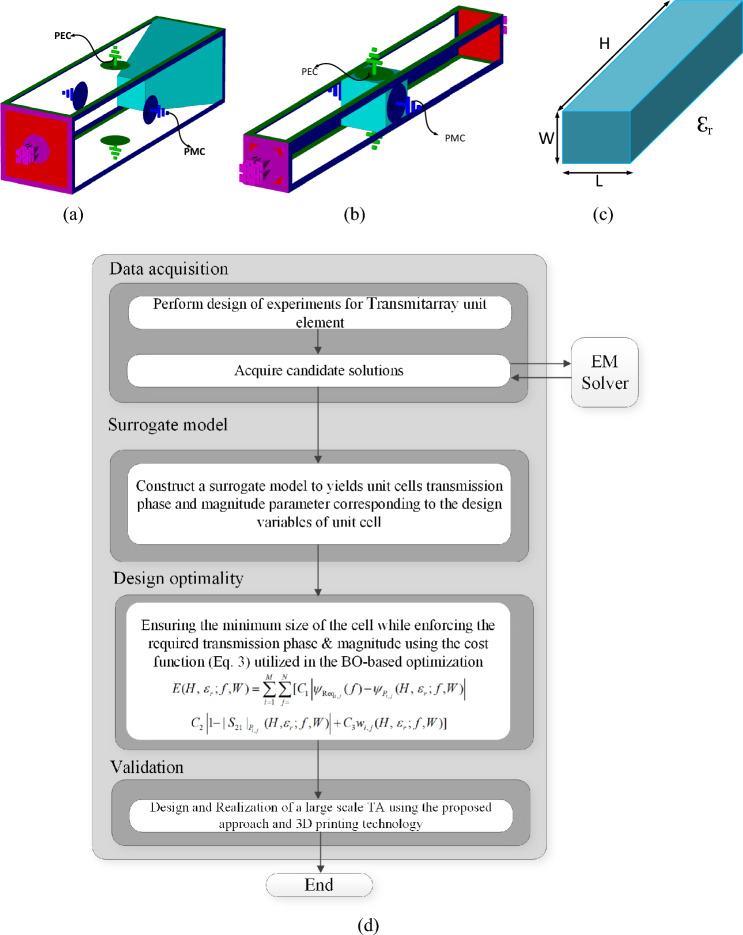
3D view of unit cell of (**a**) a pyramidal shaped reflectarray elements as a single port model ^5^; (**b**) proposed transmitarray unit cell element, (**c**) parameterization of the proposed unit element; (**d**) flow diagram of the proposed approach.

Transmitarrays ^4^ are structures inspired by reflectarray antenna designs^5^, which consist of a planar array of printed elements, and a feeding source. In contrast to the reflectarray antennas, where each unit element reflects the incoming EM waves with a certain phase (cf. Fig. 1a), in transmitarray antennas, each element manipulates the EM waves propagated from the source with the appropriately set transmission phase shift to produce a focused beam in the desired direction (cf. Figure 1b)^6,7^. Consequently, the main difference between reflectarrays and transmitarrays is analogous to the difference between mirrors and lens structures. The advantage of transmitarrays as compared to reflectarrays is that the former can achieve a high radiation efficiency without the feed blockage. The feed blockage is a challenging problem in reflectarray designs that can only be solved using complex dual-optics antennas or with offset feed configurations^8^. Despite the aforementioned advantages, transmitarray designs also exhibit several drawbacks. One of the most serious issues is a limited bandwidth, which usually does not exceed 5%; its extension can be achieved via complex multilayered element designs^9^. Further, in reflectarray designs, due to the PEC ground layer, the reflection magnitude is close to 0 dB^10,11^, so that the designer only needs to calculate the reflection phase of each element, thus the unit element can be considered as a single port model. Whereas, in transmitarray designs, on the top of controlling the phase, the transmission magnitude needs to be close to 0 dB to ensure high aperture efficiency, which makes the unit element design a two port design, which can be clearly seen from Fig. 2a,b. Consequently, design of transmitarrays is a more challenging task, which requires a manipulation of both the magnitude and the phase of the unit elements. In particular, each unit element in the transmitarray should be designed to obtain the required transmission phase delay, and the highest possible transition magnitude at the same time^12^. This can only be achieved through multi-objective optimization, which is another challenging problem due to the large number of adjustable parameters, as well as the necessity of handling conflicting requirements.

Development of transmitarrays in the sense mentioned above requires the employment of numerical optimization techniques. Unfortunately, this is computationally expensive whenever full-wave electromagnetic (EM) models are used for system evaluation. Although there are novel synthesis techniques for design optimization of lens structures which demonstrate promising results^13,14^, in the case of array designs, including transmitarrays and reflectarrays, conducting the optimization process directly at the level of full-wave EM models is normally infeasible. Even if the computational model is set with the medium mesh density, the process might take months or years to complete^15^.

A possible way of accelerating numerical optimization is the employment of data-driven surrogate models for representing the unit elements of transmitarray (cf. Fig. 2d). Fast surrogates can be used to determine—at negligible cost—the geometry and material parameters of the elements that ensure the required transmission phase and magnitude characteristics, as well as the distance between the unit element and the feed antenna^16,17^. The literature offers the abundance of surrogate-assisted methods, oriented towards improving both the computational efficiency and reliability of the optimization processes^18–24^. Still, data-driven surrogate modelling is a challenging problem in the context of high-frequency design, primarily due to the high initial cost related to the acquisition of the training and testing data sets. For transmitarray unit elements, the numbers of required samples are typically 4000 and beyond^25–27^.

This paper proposes an alternative surrogate-assisted approach to design of high-gain broad-operating bandwidth transmitarray antennas. The presented methodology is based on data-driven surrogates of unit elements of a non-planar 3D printable transmitarray, constructed by means of Artificial Intelligence (AI) techniques, which recently had shown great achievement in design optimization of Reflectarray designs^5^. Herein, instead of modeling of a single port model for characterization of S_11_ reflection phase, the handled transmitarray unit element design is a two port model where not only transmission phase but also the transmission magnitude must be included in the modelling process. Our methodology permits a rendition of reliable models using small training data sets, which translates into low initial cost of the surrogate modelling process. In particular, utilization of automated deep learning method allows for obtaining a reliable model of the unit element with only 270 training and 100 hold-out samples acquired from the EM model, which corresponds to less than one full-wave analysis of the entire array. For the sake of illustration, the presented technique is applied to design of three transmitarrays operating in the frequency bands 8–14 GHz, 22–28 GHz, and 28–36 GHz, respectively. The numerical results are validated experimentally.

The originality and the technical contributions of this work can be summarized as follows: (i) the development of a novel approach to low-cost surrogate modeling of transmitarray unit cells, (ii) demonstration of the applicability of the presented technique for constructing re-usable models that can be employed for design of TAs operating at various frequencies within the range 1–40 GHz, (iii) demonstrating computational efficiency of the modeling process and its superiority over state-of-the-art data-driven methods, (iv) demonstration the design utility of the developed model using several examples of TAs with numerical results supplemented by experimental validation of the selected array.

## Transmitarrays: simulation-driven design and challenges

As mentioned earlier, transmitarray antennas are similar in terms of the constituent elements to reflectarrays. The fundamental difference is that instead of the reflection phase manipulation in reflectarray, transmitarrays manipulate the transmission phase and magnitude to focus the EM wave, cf. Fig. 1. The analysis of transmitarray assumes that the unit elements are placed in the far-field region of feed antenna^1^. Under this assumption, the EM field incident to each unit element at a certain angle can be locally considered a plane wave, with the phase proportional to the distance from the phase center of the feed source to the element, corresponding to the spherical wave propagation^1^. In order to compensate for the spatial phase delay due to the distance between the feed and the unit element, each element in transmitarray must be carefully designed to satisfy the required transmission phase delay. Ensuring appropriate phase distribution allows for achieving a focused beam at the requested direction (Fig. 1c).

The transmission phase *ψ*_*i*_ for the *i*th element can be obtained as follows^10^:

1 $$ \psi_{i} = k(R_{i} - \vec{r}_{i} .\hat{r}_{0} ) + \psi_{0} $$

where $k$ is the propagation constant in free space, *R*_*i*_ is the distance between the *i*th element and the feed, $\vec{r}_{i}$ is the position vector of the *i*th element, and $\hat{r}_{0}$ is the target main beam direction. For a main beam in the broadside direction, the product $\vec{r}_{i} .\hat{r}_{0}$ must be equal to zero; *ψ*_0_ is a constant phase that indicates that a relative transmission phase rather than the absolute one is required for transmitarray design^1^.

Although transmitarrays exhibit the advantages of high gain and no feed blockage as compared to reflectarrays, they also have certain disadvantages. These include a limited bandwidth of up to 5%, and more involved design process that requires a manipulation of both the transmission phase and magnitude. Computationally-efficient and reliable design optimization of high-gain and wideband transmitarrays (TAs) requires addressing the following challenges:

- The ability to evaluate the transmission magnitude and phase of unit elements in a reliable, accurate, and fast way;
- The ability to improve the bandwidth of the TA elements without reducing their phase range^28^;
- The ability to manipulate both the transmission magnitude and the phase of the unit element, in particular, to maintain the transmission magnitude close to one, while ensuring wide range of transmission phase variation.

## Low-cost surrogate modeling of unit elements

In this section, we explain the unit element modeling techniques for transmitarray design. Section “Unit element modeling: methods and challenges” highlights the modeling task and the associated challenges. Section “Transmitarray unit cell and its properties” presents the 3D printable unit element considered here for TA design. Finally, Sect.”Proposed surrogate modeling approach” presents the proposed surrogate modeling framework based on deep learning with automated architecture determination through Bayesian optimization.

### Unit element modeling: methods and challenges

As mentioned earlier, transmitarrays offer an alternative way of realizing high gain antennas, which has a number of advantages over traditional methods, as indicates in Sect. “Introduction”. In transmitarray antennas, each element manipulates the EM waves propagated from the source with the appropriately set transmission phase shift to produce a focused beam in the desired direction. To achieve the desired performance, the unit elements of transmitarray antenna must meet certain criteria as mentioned in Sect. “Transmitarrays: simulation-driven design and challenges”.

In the literature, different types of unit elements have been proposed for implementing high-gain transmitarrays. Examples include multi-layer TA designs with dipole elements and seven conductor layers^5^, or double-square-loop element with four conductor layers^9^, achieving 360 degree of phase manipulation. It should be noted that although it is possible to increase the bandwidth of the TA using multi-layer designs, this approach also increases the thickness and the transmission loss, and, consequently, the weight and the manufacturing cost^9^. More complex unit element deigns such as Jerusalem-cross shapes^7^ as are also taken into consideration to reduce the total number of layers at the expense of reduced phase manipulation range of 335 degrees. As mentioned earlier, to reduce the design cost and complexity, the primary challenge is to achieve a full phase range of 360 using a smaller number of conductor layers, while avoiding the reduction of the element transmission magnitude, and maintaining the overall performance of the transmitarray antenna^4^.

Thus, design of transmitarrays is a challenging endeavor, which requires careful determination of geometrical parameters of each unit element within the TA, so that both the required transmission phase delay, and the highest possible transmission magnitude is simultaneously achieved^12^. A proper approach is multi-objective optimization, which is an intricate procedure by itself, not only due to the large number of variables, and the necessity of handling conflicting requirements, but also due to the extremely high cost of evaluating TA characteristics, which involves full-wave electromagnetic analysis^13^.

To work around direct handling of expensive EM models, surrogate-assisted optimization has been studied by many researches over the last decades. The literature offers a number of algorithmic tools in the context of reflectarray and transmitarray design (e.g.,^22–24^). Notwithstanding, the high initial cost of surrogate model construction, pertinent to the acquisition of the training and testing data samples (typically, 4000 samples or beyond^25–27^) is still a major practical issue.

### Transmitarray unit cell and its properties

The proposed work is a multidisciplinary work involving microwave antenna theory (Electrical Engineering/Energy) and artificial intelligence (Computational Science) each of which are extremely complex topics. In order to present this topic for all the possible readers’ authors aimed to firstly present the concept of artificial intelligence on a simple TA unit element such as example given in Fig. 2 which can also be modelled using analytical formulations^29^. The fundamental purpose of this example is to present how one should select the design variables with respect to the EM response and how to can be applied on AI models, whereas the second and the third examples provide more challenging design cases that can only be solved via EM simulations and AI techniques. Thus, by this mean, authors aim to illustrate the presented concepts and workflows using simple test case, so that the challenges pertinent to the examples themselves do not make demonstration of the concepts unclear. The modeling technique proposed in this work is accompanied by an exemplary unit element with variable length and relative dielectric constant. This arrangement makes it suitable to satisfy the following requirements: (i) wide range of variation for the transmission phase, (ii) low loss transmission medium for EM signals, (iii) easy fabrication via 3D printing, (iv) broad range of achievable relative dielectric constant values, which can be adjusted between 1.3 and 2.7^30^. The dielectric properties are controlled by the infill rate of material density used during the 3D prototyping, which can also reduce the overall weight of the design. The cell architecture, shown in Fig. 2b–c, is modeled through full-wave EM analysis (here, using CST MWS). The top and the bottom surfaces of the 3D model are perfectly electric conducting walls, whereas the right and the left walls are perfectly magnetic field walls^11^. The incoming waves will be incident normally onto the element, and transmitted to the second port with a variable transmission phase depending on the length and the relative dielectric constant of the unit element. The length of the E and the H walls of the computational domain, and the position of the waveguide ports are fixed. The range of design variables are taken as 1.9 ≤ *ε*_*r*_ ≤ 2.7, 2 ≤ *H* ≤ 60. The simulation frequency range is from 1 to 40 GHz. The cell width and length are set to *W* = *L* and taken in the range of 10 ≤ *W* ≤ 20 mm for sensitivity analysis. It should be mentioned that although *W* has a limited effect on both the phase (< 2°) and the magnitude (< 0.01) of the unit cell transmission response, this parameter is important for array design as it determines the overall weight of the structure. Commonly, *W* is taken as equal to *λ*_0_/2 (half of the free-space wavelength)^4^, due to wide range of applicability of the unit element (1 ≤ *f*_*r*_ ≤ 40 GHz) this value should also be included in the optimization process of transmitarrays weight for the selected operating frequency band.

**Figure 3:**
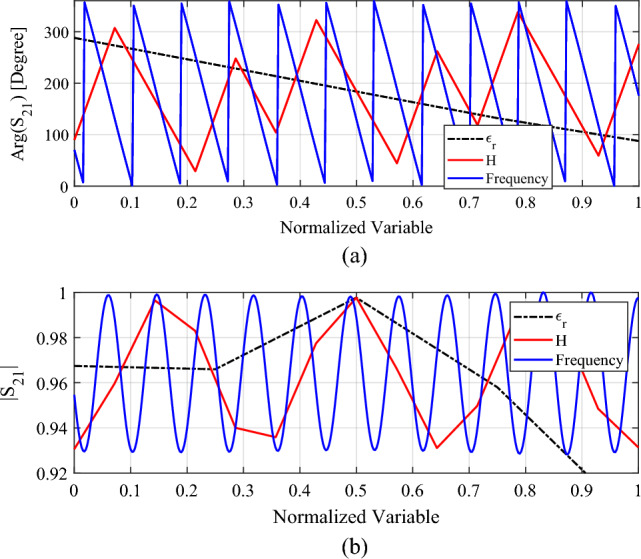
Parametric analysis of the unit cell’s transmission: (**a**) phase, (**b**) magnitude as a function of the normalized variables. Here, each of the variables *ε*_*r*_ [1.9 ~ 2.7], *H* [2 ~ 60] and frequency [1 ~ 40 GHz] are normalized between 0 and 1 to show their effects on the transmission characteristics of the unit element. During the parametric analysis of each variable, the other parameters are fixed as follows: *ε*_*r*_ = 2.1, *H* = 30, frequency = 20, *W* = *L* = 15 [mm].

In summary, each of the variables *ε*_*r*_ , *H, W, f*_*r*_, has a unique effect not only on the transmission characteristic but also the weight of the cell. Again, as the effect of *W* on the transmission characteristics of the unit cell is almost negligible, this parameter will not be incorporated into the surrogate model of the cell. Consequently, their determination is the main challenge of this study, as elaborated on in Sect. “Results and experimental validation” Figure 3 shows a parametric analysis of the effects of unit cells parameters on the transmission phase response. It can be observed that a broad range of transmission phase variation is achieved, thus the designed unit cell is a suitable candidate for design of high performance transmitarrays.

Table 1 illustrates the effects of the unit cell parameters on its transmission characteristic. A broad range of phase variations can be observed, which is sufficient for transmitarray design purposes. However, it should be emphasized that the design process has to account for additional factors: (i) the lengths of adjacent cells cannot be significantly different from each other to avoid a blockage for the incoming EM waves at the corners or distanced locations of the array; (ii) increasing both the length and the relative dielectric of cell can increase the variation range of transmission phase; however, it would also increase the overall weight and the manufacturing cost of the design. The latter can be calculated by multiplying the unit cell volume by the material density, *w*_0_ = *ρV*.

**Table 1:** Parametric analysis of the proposed unit element: transmission characteristics for different geometry and material parameter setups (W = L = 15 [mm]).

Cell parameters			S_21_			
*ε*_*r*_	*H *[mm]	Frequency [GHz]	Real	Imaginary	Magnitude	Phase [Degree]
1.7	30	20	− 0.723	0.666	0.988	137.3
2.1	30	20	0.802	0.559	0.977	34.8
2.7	30	20	− 0.228	− 0.863	0.892	− 104.8
2.1	2	20	0.282	− 0.898	0.941	− 72.5
2.1	30	20	0.802	0.559	0.977	34.8
2.1	58	20	− 0.701	0.672	0.971	136.2
2.1	30	1	− 0.342	− 0.891	0.954	− 111.0
2.1	30	20	0.802	0.559	0.977	34.8
2.1	30	40	0.482	0.785	0.921	58.4

The transmitarray is to be manufactured using the 3D printing technology. Here, it should be noted that the infill rate of printed design will directly affects the relative dielectric constant of the structure^30^, which can be used for changing the transmission phase, magnitude, and the weight of the design.

Further, there are additional parameters that can affect the overall weight of the design, such as the minimum number of solid layers for both ground and top layer of the 3D printed structure, the layer height precision, and material used. The following formula, obtained from the data presented in^5^, allows for calculating the overall weight of a unit cell prototyped by using PLA material:

2 $$ w = 1.276 - 0.321w_{0}^{{}} - 1.235\varepsilon_{r}^{{}} - 0.00542w_{0}^{2} + 0.491w_{0}^{{}} \varepsilon_{r}^{{}} + 0.287\varepsilon_{r}^{2} $$

where *w*_0_ is the weight of a design with 100% infill rate, whereas *ε*_*r*_ is the target dielectric constant of the unit cell. The latter is directly related to the infill rate.

### Proposed surrogate modeling approach

For most engineering problems, the relationships between the design parameters and the system characteristics are nonlinear. One of the common methods for modelling nonlinear data are Artificial Neural Networks (ANNs)^31,32^. A Multi-Layer Perceptron (MLP) is one of the most well-known and favored ANN architectures^33^. The main reasons include the ability to model multiple outputs at same time, and to transform the modelling space to a higher dimension by using hidden layers and neurons’, which make this method an ideal technique for creating a mapping between the design parameters and the system characteristics. However, in order for MLP to create an accurate model for highly nonlinear systems, the overall complexity, the hidden layer size, and total number of neurons, of the model must also be increased. This leads to one of the major drawbacks of MLP, which is vanishing gradient^34^. One of the efficient methods to mitigate this issue is the employment of more involved Deep Neural Network (DNN) algorithms^35^.

With the usage of modern activation functions such as ReLU^36^, LeakyReLU^37^, and the normalization layers, the vanishing gradient problem or the saturation of neuron weighting coefficient can be prevented. A hybrid model between the traditional MLP and the state-of-the-art DNN models is Modified Multi-Layer Perceptron (M2LP)^38^. Another advantage of M2LP, being a result of its hybrid structure, is its intra-space and inter-space transformation capability. In the sub-layer formation of M2LP, the data is handled by two hidden layers of identical neuron sizes to create an initial mapping of the input data, which is referred to as the intra-space transformation. The inter-space transformation is formed between the output and the input of the two sub-layers where the number of hidden neurons is increased twice as compared to the preceding layer. Based on these transformations, M2LP is able to represent complex relationships, which facilitates handling of nonlinear characteristics. Finally, the M2LP an expansion layer is applied to make the model space compatible with the size of the system outputs.

**Figure 4:**
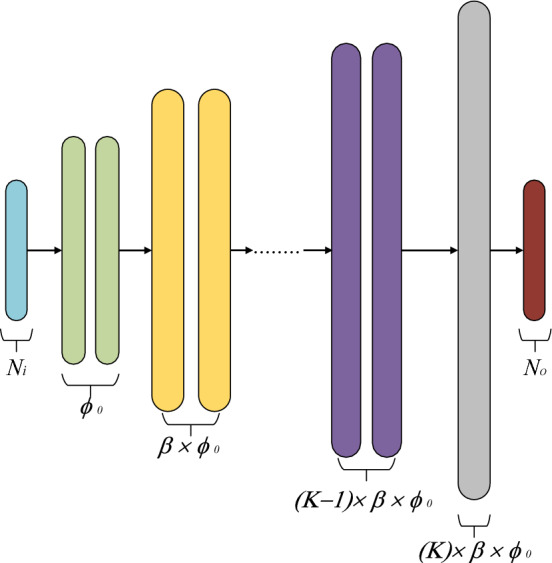
General architecture of the proposed M2LP model. The user only defines the number of sub-layers *θ*, and the multiplication factor *β* that controls the number of neurons in each interspace transformation. Here, these values are taken as *θ* = 2, and *β* = 2, respectively. All other parameters are determined through optimization.

Figure 4 shows the architecture of the M2LP model. In each of the blocks an intra-space transformation based on two layers (of same color in the picture) is performed for three sub-layers (marked as green, purple, and orange, respectively). The last layer is the expansion layer (grey). The blue and the red layers represent the inputs (*ε*_*r*_, *H, W, f*_*r*_) and the outputs (|S_21_|, ∠S_21_,) of the system at hand.

It should be noted that appropriate determination of the model hyper-parameter is instrumental in ensuring the best possible predictive power of the surrogate model. In this work, automated architecture determination of M2LP is carried out using Bayesian Optimization (BO) ^39^. The optimum hyper-parameter setup of M2LP is obtained using a search space defined as follows: (i) initial number of neurons *ϕ*_0_ ∈ {16, 32, 64, 128, 256, 512}, (ii) the number of blocks *K* ∈ {2, 3, 4, 5}, (iii) the leakage parameter of LeakReLU activation function 0.05 ≤ *α* ≤ 0.25.

The number *θ* of sub-layers and the factor $\phi$ controlling the increase the number of neurons in the layers is assumed to be fixed and determined by the user. A *k*-fold cross-validation with *k* = 3 is being to guide the optimization process using the training data that consists of 270 samples. Furthermore, an additional (hold-out) data set with 100 samples (randomly selected) is used to evaluate the over-fitting performance of the model. Here it is worth mentioning that the total computational cost of the surrogate modelling approach will be calculated based on both training and hold-out data sets since all the data are employed in the modelling process. Each data sample is a vector of the size 1 × 391 representing the evaluation of the unit cell within the frequency band of 1–40 GHz which is uniformly distributed with step size of 0.1 GHz.

### Modeling results and benchmarking

One of the main contributions of this work is a computationally efficient data-driven surrogate modeling with a reduced the number of training data samples required for constructing a globally accurate data-driven surrogate for a TA unit cell. Here, it is noteworthy that the major contribution to the computational cost of data-driven surrogates is the acquisition of the training and testing data samples (typically, 4000 samples and beyond^25–27^). Reducing this number is challenging yet imperative for improving the computational efficiency of the model process. In this work, by using the modelling approach proposed in Sect. “Low-cost surrogate modeling of unit elements”.*C*, an accurate surrogate model of TA unit cell is obtained only using 370 data points, obtained using a linear sampling technique with the step sizes of 6, 3, 15, for *ε*_*r*_, *W, H* respectively. For the sake of benchmarking, the proposed M2LP modelling approach is compared to the methods commonly used in the context of surrogate modelling of similar microwave antenna designs: (i) Support Vector Regression ^19,24,40^, (ii) Ensemble Learning^41^, and (iii) Generalized Regression Neural Network (GRNN)^42^. The hyper-parameter configuration of the considered methods are presented in Table 2. Table 3 gathers the modeling error values for all surrogates based on performance results of hold-out data set.

**Table 2:** Hyper-parameter configuration of surrogate models (benchmark and the proposed one).

Model	Model name	Model specifications
1	Lib SVM ^43^	Tolerance of termination: 1e-4
		Cost: 1e-3, SVM: epsilon, Kernel: radial
2	Ensemble Learning	Learner type: LSboost, cycles: 1000
		Learning rate: 0.091, Number of splits: 750
3	GRNN	Spread parameter: 0.46
4	M2LP (this work)	Initial learning rate: 10^–2^ , Maximum epoch: 500, Mini batch size: 25% of data set, Optimizer: ADAM

**Table 3:** Modeling results and benchmarking.

Parameter	Mean absolute error			
	SVRM	GRNN	Ensemble Learning	M2LP (this work)
|S_21_|	0.019	0.024	0.022	0.017
∠S_21_	17.6	30.4	26.8	5.7

**Figure 5:**
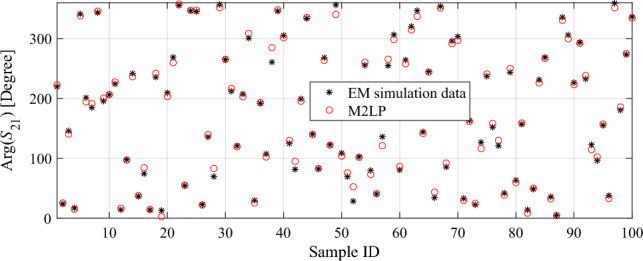
The proposed M2LP surrogate versus EM simulation data for ∠S_21_, using 100 randomly selected data points from the holdout dataset.

As it can be seen from the results provided in Table 3, although all the models exhibit high accuracy in representing |*S*_21_|, this is mainly due to the linearity and small range of variation of this response. The proposed M2LP regression model ensures a significantly better MAE value of 5.7 for the transmission phase (Fig. 5), which is the most important response from the perspective of TA design. At the same time, the benchmark methods exhibit at three to six time’s higher error. Among these, only the Support Vector Regression surrogate is capable of achieving MAE of less than twenty degrees for the transmission phase, whereas the errors of Ensemble Learning and GRNN are as high as 26.8 and 30.4, respectively. In other words, the modeling approach presented here allows us to ensure sufficient performance even when using sparse data sets.

At this point, it should be emphasized that although SVRM and other benchmark surrogates have been demonstrated successful in modeling of different microwave components^19,24,40,44^, the results presented above suggest otherwise in the context of transmitarrays. The main reason for this is that performance of SVRM and other benchmark surrogate modeling techniques is contingent upon the variation ranges of the input parameters (which are normally set rather narrow), and the total number of samples (270 + 100) to generate a globally accurate surrogate model^5,45^. If the parameter ranges are restricted or the training dataset cardinality is sufficient, such as shown in the case of modelling the modulus of the transmission characteristic, most of surrogate modelling methods will perform well^5,45^. However, the objective of this work is to construct design-ready models suitable for TA development using small datasets (only 370 samples). As demonstrated, this goal is beyond the capabilities of popular models such as SVRM and other traditional approaches. In contrast to this, the proposed M2LP-based surrogate ensured the accuracy sufficient for design purposes owing to its state-of-the-art configuration and utilization of modern activation functions inherited from deep learning methods, both improving its handling of the non-linear relationships between inputs and outputs of the problem. The M2LP model will be used in the next section for design optimization of a large-scale transmitarray designs targeting three different operation frequency ranges.

## Results and experimental validation

This section demonstrates the employment of the M2LP unit cell surrogate model discussed in Sect. “Low-cost surrogate modeling of unit elements” to develop a large scale transmitarray. The design goals include optimization of all array cells with respect to their geometrical design parameters, *H*, *W* and ε_*r*_, so as to achieve the required transmission phase and the maximum magnitude characteristics, but ensure a possibly low weight *w*, as defined by (2). The cost function utilized in the BO-based optimization of *M* × *N* transmitarray has been formulated as follows

3 $$ \begin{gathered} E{(}H{, }\varepsilon_{r} {; }f,W{)} = \sum\limits_{i = 1}^{M} {\sum\limits_{j = }^{N} {[C_{1} \left| {\psi_{{{\text{Req}}_{i,j} }} (f) - \psi_{{P_{i,j} }} {(}H{, }\varepsilon_{r} ;f,W{)}} \right|} } \\ C_{2} \left| {1 - |S_{21} |_{{P_{i,j} }} {(}H{,}\varepsilon_{r} ;f,W{)}} \right| + C_{3} w_{i,j} {(}H{, }\varepsilon_{r} ;f,W{)}] \\ \end{gathered} $$

where $\psi$_Reqi.j_ is the required transmission phase at the operating frequency *f*, $\psi$_*Pi,j*_ is the transmission phase predicted by data driven surrogate model. The second term in the Eq. (3) is to ensure that the unit element achieves maximum transmission magnitude alongside with the requested phase. *w*_*i.j*_ is the weight of the unit element calculated according to (2), whereas *C*_1_ and *C*_2_ are the weighting coefficients representing the relative importance of the phase- and weight-related objectives, respectively. Here, we set *C*_1_ = 0.7 and *C*_2_ = 0.3, and *C*_3_ = 10, these values are taken with respect to the variation limits of each aimed criteria to obtain a balanced effect of each term on the cost function. For example, since the numerical values of the transmission phase may be larger than 100, and the weight of the unit element is usually around 2 g, the weighting factors corresponding to these terms are set lower than unity. Whereas, the magnitude of |*S*_21_| is smaller than one, therefore, its corresponding weighting factor is set at a larger value.

In order to demonstrate the design utility of the surrogate model developed in Sect. “Transmitarrays: simulation-driven design and challenges”, it is used along with the cost function (3) to design TAs operating at different frequency ranges: (I) 8–14 GHz, (II) 22–28 GHz, (III) 28–36 GHz. Each of these designs consist of 20 × 20 unit elements, whose parameters are optimally selected via BO-assisted data driven surrogate models. For each of the array elements, BO will make a separate search to determine the optimal values of *H*, *W* and *ε*_*r*_, so as to obtain the required transmission phase $\psi$_Req*i.j*_, the latter calculated using (1)^10^, and the transmission magnitude |*S*_21_|. The aim of surrogate-assisted BO search is to minimize the error function (3). It should be emphasized that in either of reflect or transmit array designs, the average phase error of ± 10 degrees in each element can be taken as an acceptable tolerance level for having designs with high directivity characteristics^46,47^. This means that the accuracy of the surrogate model, especially the part representing the transmission phase, plays a critical role. At the same time, using small datasets reduces the computational cost of training data acquisition, thereby improving the efficacy of the design process. If the surrogate model fails to ensure a sufficiently good predictive power, the resulting TA design would suffer considerable gain loss^48^.

The specific designs considered in this work are 20 × 20 arrays, aimed to operate at the aforementioned frequency bands. To simplify the optimization process, all TA designs are assumed to be symmetrical in both the *x* and *y* axis, so that the array can be divided into 4 parts consists of 10 × 10 elements. Furthermore, each of the quarters can also be considered diagonally symmetrical. Under this assumption, the total number of elements that have to be optimized is 55. Also, *ε*_*r*_ of the design is taken constant for all of the unit elements in a range suitable from the point of view of prototyping by means of 3D printers, and the PLA material^49,50^. However, it is worth mentioning that if a high-end professional 3D printer is available to designer, it is possible to prototype a non-homogeneous array where each element might have individual infill-rate, consequently allowing each unit cell to have a unique dielectric constant value. However, the prototyping device used in this work only allows a single value of infill rate in the prototyping process.

In this work, to unambiguously present the importance of the accuracy of data-driven surrogate models, the TAs were designs using the proposed M2LP-based model, and the best of the benchmark techniques, SVRM. The BO is selected as the optimization search protocol. It should be noted that although the literature offers a plethora of meta-heuristic^51,52^ or gradient based^53,54^ optimization algorithms, the particular choice of the algorithm is not critical here because the optimization process is conducted at the level of fast and analytically tractable metamodel. Even massive evaluations thereof would incur negligible computational expenses. Consequently, the performance measures such as speed or the convergence rate of the algorithms to the global optimum are not in studied in this work.

**Figure 6:**
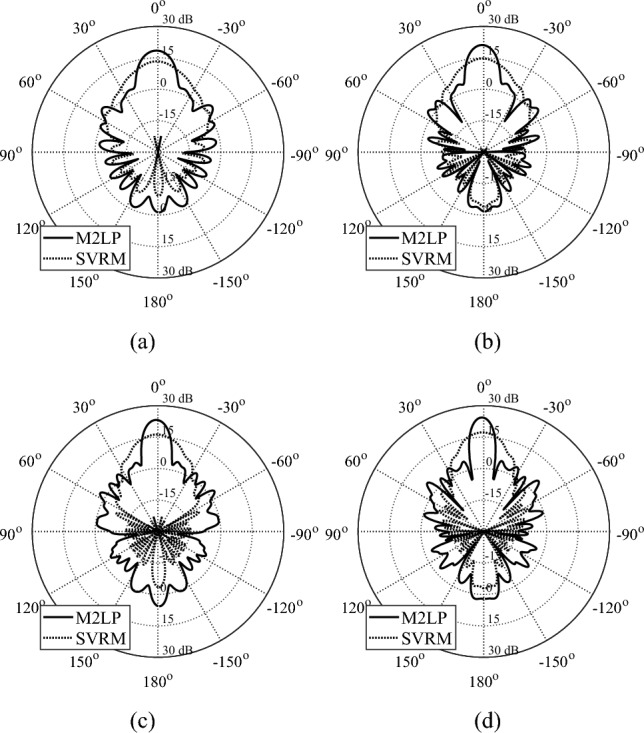
Optimized TAs obtained using M2LP and SVRM. EM-simulated realized gain of designs at (**a**) 8 GHz, (**b**) 10 GHz, (**c**) 12 GHz, (**d**) 14 GHz. Note a significantly better performance of the array developed using the proposed M2LP surrogate over the one obtained with the SVRM model.

**Figure 7:**
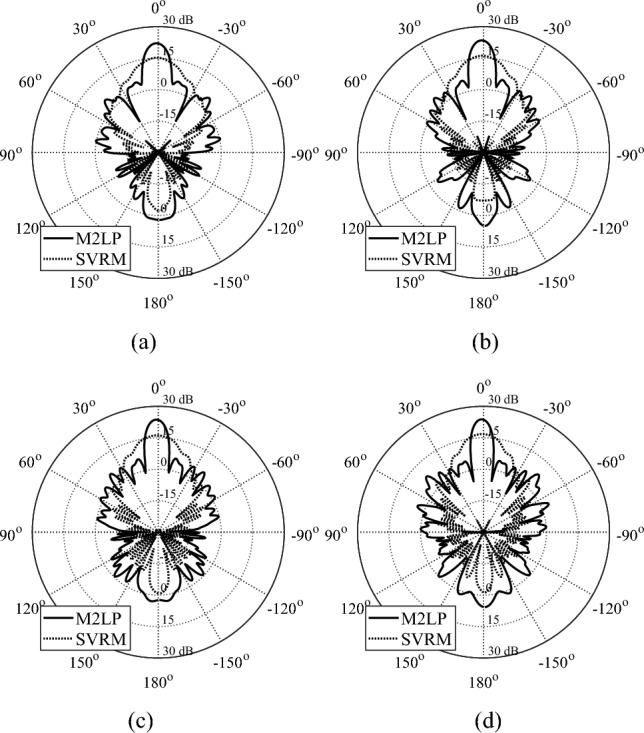
Optimized TAs obtained using M2LP and SVRM. (**a**) 22 GHz, (**b**) 24 GHz (**c**) 26 GHz, (**d**) 28 GHz.

**Figure 8:**
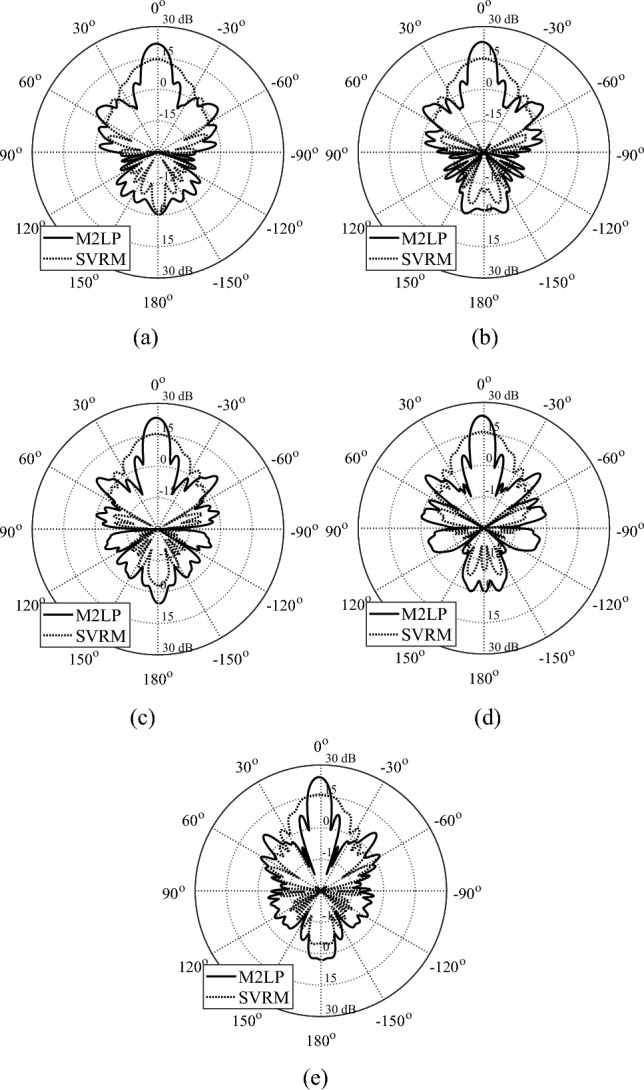
Optimized TAs obtained using M2LP and SVRM. (**a**) 28 GHz, (**b**) 30 GHz (**c**) 32 GHz, (**d**) 34 GHz, (**e**) 36 GHz.

The optimization outcome for both surrogates has been presented in Table 4. The cost value of M2LP models and SVRM are similar thus the overall performance of the obtained models expected to have similar performance characteristics. However, as the accuracy of SVRM is significantly worse than that of the proposed M2LP surrogate, the actual performance of the respective TAs is dramatically different. As shown in Figs. 6, 7 and 8 (simulated radiation patterns) and Table 5 (gain and side lobe levels), the designs obtained using SVRM exhibit significant performance loss. Given similar cost function value in Table 3, the sole reasons for this is the huge performance gap between the accuracy of the models (absolute phase error of 5.7 degrees for M2LP versus 17.6 degrees for SVRM).

**Table 4:** Design specifications of M2LP- and SVRM-based TA designs.

Design	Used Surrogate	Design specifications	
		Total cost value	Total weight [Gram]
I	M2LP	0.73	391
	SVRM	0.71	378
II	M2LP	0.68	48.1
	SVRM	0.69	49.2
III	M2LP	0.77	22.7
	SVRM	0.81	23.2

**Table 5:** Simulated performance of M2LP- and SVRM-based TA designs.

Design	Array characteristics		Surrogate model used	
	Performance figure	Frequency [GHz]	M2LP	SVRM
I		8	18.4/-14.7	13.3/−12.5
	Maximum Gain [dBi]/Side Lobe Level [dB]	9	19.9/-16.5	14/−13.5
		10	21.1/-16.7	14.7/−13.8
		11	22.5/-17	15.9/−15.6
		12	23.2/-17.6	16.3/−15.4
		13	23.5/-19.7	16.8/-17.1
		14	24.4/-18.9	17.3/−17.8
II		22	22.1/-16.6	15.2/−12.3
		24	23.5/-17.7	16.2/−13.5
		26	23.7/-17.7	16.2/−12.9
		28	23.8/-15.9	16.8/−14.1
III		28	21.8/-16.4	14.4/−11.4
		30	22.7 7 -16.9	14.8/−11.1
		32	23.1/-15.5	15.5/−11.6
		34	23.7/-16.5	16/−12.2
		36	24.2/-16.3	15.9/−11.3

In Table 6, computational performance comparison of the proposed surrogate modeling approach and the Forward Electromagnetic (FW-EM)-based model is presented. The total cost of the proposed approach corresponds to 370 unit cell simulations necessary to obtain the training and hold-out points for generating the surrogate model using the FW-EM simulation model (20 s). The optimally-designed TA, which consist of 20 × 20 = 400 elements is obtained by optimizing 55 unit elements (due to the symmetrical placement of the elements) using surrogate-assisted Bayesian Optimization (set to a computational budget of 50 iterations), and an additional single run of FW-EM tool for the entire array implementing the optimized design. Based on the results of Table 6, it can be stated that the proposed surrogate modeling approach enables a significant acceleration to the design optimization process as compared to the traditional FW-EM optimization approach. More specifically, the proposed method is almost 100 times faster (267 h vs. 2.85 h). It should also be reiterated that from the method presentation perspective, the specific array designs considered here are merely illustration examples.

**Table 6:** Performance comparison of the proposed approach and FW-EM-model-based design in terms of the computational cost of individual simulations and total design process. The simulations have been done using the following simulation setup: AMD Ryzen 7 3700X 8-Core Processor 3.59 GHz, with 32.0 GB of installed RAM, and NVidia 2080 GPU 8 GB.

Model	Model specifications	Simulation time
Single unit element in FW-EM simulator	Cells per wavelength & max model box edge = 15	~ 20 [Seconds]
	Fraction of maximum cell near to model = 20	~ 2.0 [Hours] for 370 samples
	Mesh size = 2700	
Training of the M2LP model	BO algorithm based search with iteration of 30 for given hyper parameters in section (Proposed Surrogate Modeling Approach) with model specifications in Table 2	~ 0.5 [hours]
Single run of surrogate model	The surrogate model is generated using 370 cells using FW-EM model	0.1 [Seconds]
Single run of FW-EM tool for TA array	Cells per wavelength & max model box edge = 15	~ 16 [Minutes]
	Fraction of maximum cell near to model = 20	
	Mesh size = 4,649,908 for an array of 20 × 20 array	
FW-EM based optimization of TA	Built-in particle swarm optimization with swarm size of 50 and maximum iteration of 20, using uniform random distribution	~ 267 [Hours] (16 × 1001)
Total design cost of the proposed approach	The optimally designed TA is obtained via optimization of 55 unit elements based on surrogate model assisted BO-optimization (50 iteration) and a single run of FW-EM tool for an array of 20 × 20 array	~ 2.85 [Hours]
		2.0 Hours + 0.5 Hours + 55 × [50 × 0.1 Second]
		+ 16 Minutes

Poor accuracy of SVRM translates into inferior performance of the EM-simulated TA, both at the level of individual unit cells (surrogate prediction versus EM evaluation), and the entire system.

**Figure 9:**
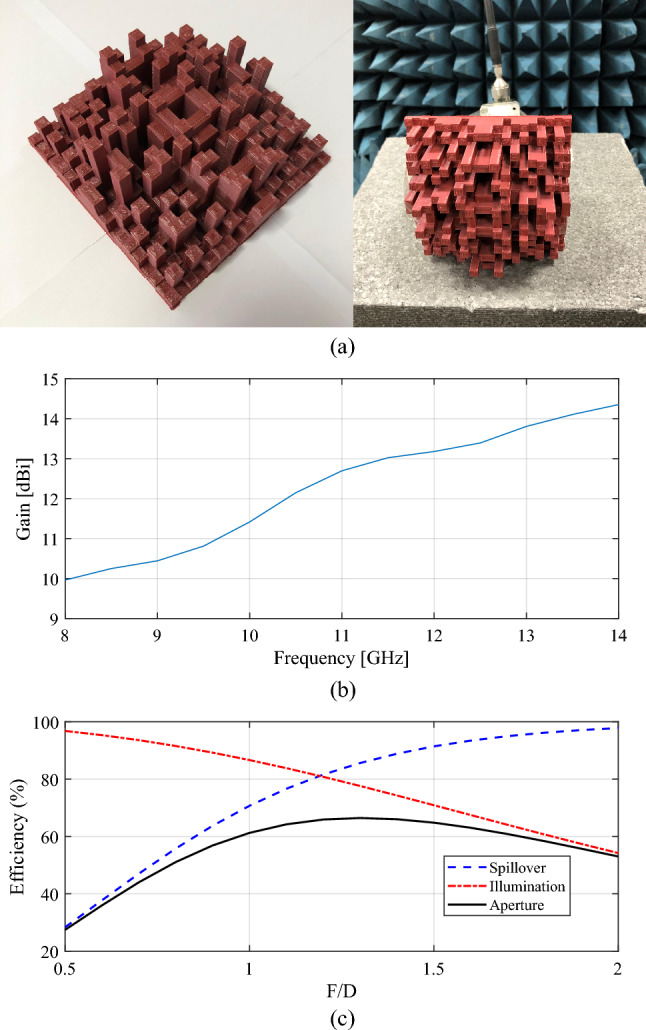
(a) Photograph of the 3D printed arrays optimized using M2LP; (b) Measured gain of the feed antenna; (c) Efficiencies of the TA design.

**Figure 10:**
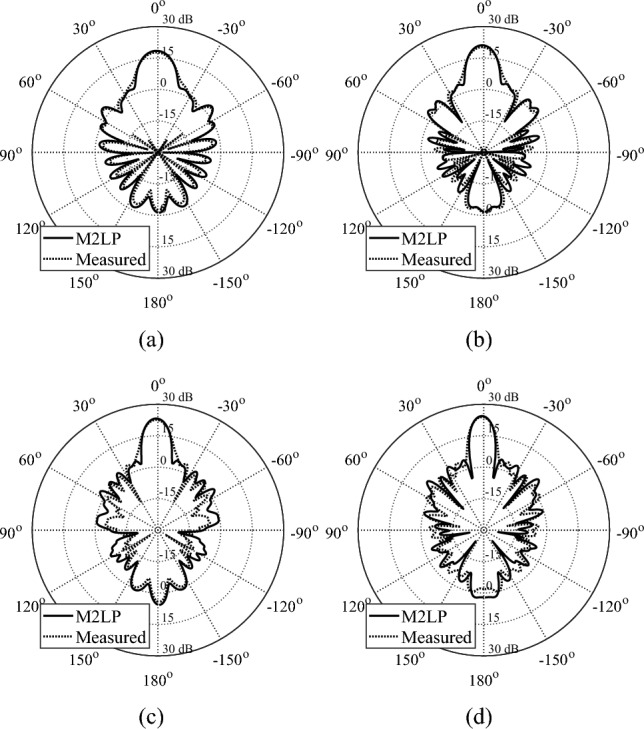
Experimental validation of the transmitarray [12 × 12 × 5] cm design: (**a**)–(**d**) measured realized gain [dBi] for: (**a**) 8 GHz, (**b**) 10 GHz (**c**) 12 GHz, (**d**) 14 GHz. Surrogate-predicted and measured data shown using solid and dashed lines, respectively.

The data in Table 5 indicates that the TA designs generated using the M2LP surrogates exhibit at least 5 dBi higher radiation gain for any of the three considered designs. For further validation of the proposed modelling approach, the TA model aimed for 8–14 GHz operation band has been manufactured, Fig. 9a, using the RoboxDual by CEL–A Dual Material 3D printer^55^ and Polylactic acid (PLA) 1.75 mm 3D printing filament^56^. A 9 kHz-to-13.5 GHz Vector Network Analyzer, and LB-8180-NF broadband 0.8-to-18 GHz horn antenna, available at Yildiz Technical University have been used for the measurement. The size of the prototyped TA is 4λ × 4λ × 1.67λ, size of the Feed antenna is 1.47λ × 1.07λ × 1.84λ, and the distance of the TA and aperture of feeding antenna is 3.87λ (λ = 3 cm for the center frequency of 10 GHz. Gain of the feed antenna is measured as10-14 dBi for the operation band of 8–14 GHz (Fig. 9b), and the illumination and spillover efficiencies of the design is presented in Fig. 9c. The simulated/measured radiation patterns of the prototyped TA at 8 GHz, 10 GHz, 12 GHz, and 14 GHz are presented Fig. 10a–d. As it can be seen from these results, the experimental data is well aligned with the simulations: the difference does not exceed 1 dBi. The aperture efficiency of prototyped design is measured based on Eq. 4, where *A*, λ, $G_{M}$ are the area of prototyped TA, wavelength at observed frequency, and measured gain.

4 $$ \eta = \frac{{\lambda^{2} G_{M} }}{4\pi A} $$

Furthermore, in Table 7, series of counterpart TA designs from literature^57–65^ are taken under study to compare the performance of prototyped TA with proposed approach. Performance measures such as operation band, size, maximum gain and aperture efficiency of each designs are taken under investigation. As a results, the proposed design achieves a good overall performance compared to its counterpart design. Here it must be empathized that the performances such as maximum gain of the TA can further improved by simply increasing the distance of the TA design from the aperture of the feed antenna and by increasing the total number of element, i.e., enlarging the design or by including other design considerations to the model such as diffraction, local periodicity assumption which here might be effected by the height difference between two neighbors, coupling effects of neighboring unit elements etc. which can affect the radiation performance of the design. On the other hand, it should be reiterated that the main purpose of the work was to introduce a surrogate modeling technique capable of providing low-cost models suitable for rapid EM-driven design optimization of transmitarray antenna designs, rather than to propose a new design of a high-performance TA. From this perspective, the specific array design is merely an illustration example. Yet, as it can be seen form the results the obtained measured results suggested that the proposed approach even with its limitations on modeling side and conflicting optimization goal of design must have lowest possible weight which would reduce the fitting (difference between desired transmission phase/ magnitude and the elements response) between optimal transmission phase and magnitude that directly effects the radiation performance, the overall performance of the design is compatible and even superior to series of counterpart design in literature (cf. Table 7).

**Table 7:** Table of comparison for different TA deigns.

Work	Center frequency [GHz]	Structure	Max gain [dBi]	1 dB gain bandwidth [%]	Aperture efficiency [%]	Size [λ]	*ε*_*r*_	Number of elements	F/D
^57^	30	Dielectric	25.1	13.3	40.3	8 × 8	–	16 × 16	1.43
^58^	14.5	Dielectric	23	29	11.0	2412.4 × 12.4	5/ 8/ 11	31 × 31	0.625
^59^	30	Dielectric	31.6	16	51.6	15 × 15	10	50 × 50	–
^60^	12	Microstrip	22.6	5.4	44.0	5.72 × 5.72	2.2/ 10.2	11 × 11	0.71
^61^	10.3	Microstrip	21.9	11.6 (3-dB)	21.8	7.4 × 7.4	3.5	17 × 17	0.49
^62^	6	Metal	19.2	NA	11.3	–	–	628	–
^63^	12.4	Microstrip	25.8	16.8	46.5	8.8 × 8.8	3.5	233	1
^64^	19.75	Microstrip	31.8	15.2	60.2	14.4 × 14.4	3.5	1613	1
^65^	20	Microstrip	25.7	–	48	7.6 × 7.6	2.2	20 × 20	–
Here	11	Dielectric	21.7	32.1	59.4	4.4 × 4.4	2.4	20 × 20	1.42

**Figure 11:**
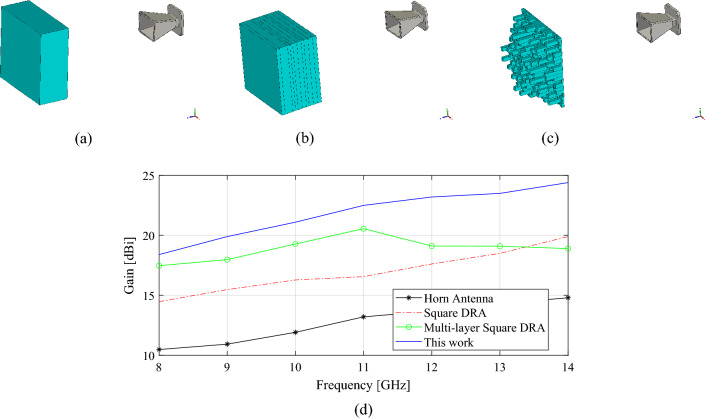
3D views of (**a**) a simple single-layer antenna, (**b**) multi-layer dielectric lens antenna; (**c**) 3D printed TA design designed using the proposed methodology, (**d**) simulated maximum gain over the frequency band.

To further illustrate the proposed methodology and its performance, a series of additional analyses are presented. First, the performance of the 3D printable antenna design is compared to a dielectric lens antenna. In Fig. 11a–d, the 3D models and simulated results of two dielectric antenna designs are presented to serve as a benchmark for the design studied in this work (Fig. 11c). For fair comparison, all considered designs are developed to feature similar sizes and configuration to the proposed TA. As it can be seen form the simulated maximum gain data, the proposed TA does not only exhibit higher gain but also its overall weight is significantly lower than that of the benchmark designs. The only disadvantage of the proposed TA design is the design complexity due to the unit cell and high number of design variables which, has been effectively addressed by the proposed methodology (specifically developed to handle design-related issues).

**Figure 12:**
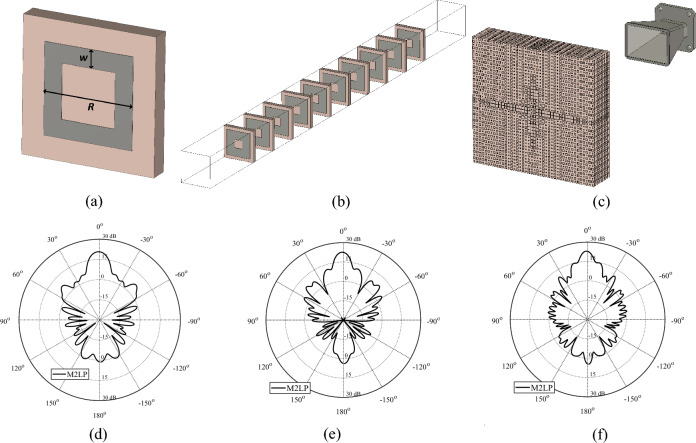
Schematic view of Model 2 unit cell, (**b**) 10 layers of identical unit cell of Model 2; (**c**) optimized Model 2 TAs obtained using M2LP: EM-simulated realized gain of designs at (**d**) 8 GHz, (**e**) 10 GHz, (f) 14 GHz.

**Figure 13:**
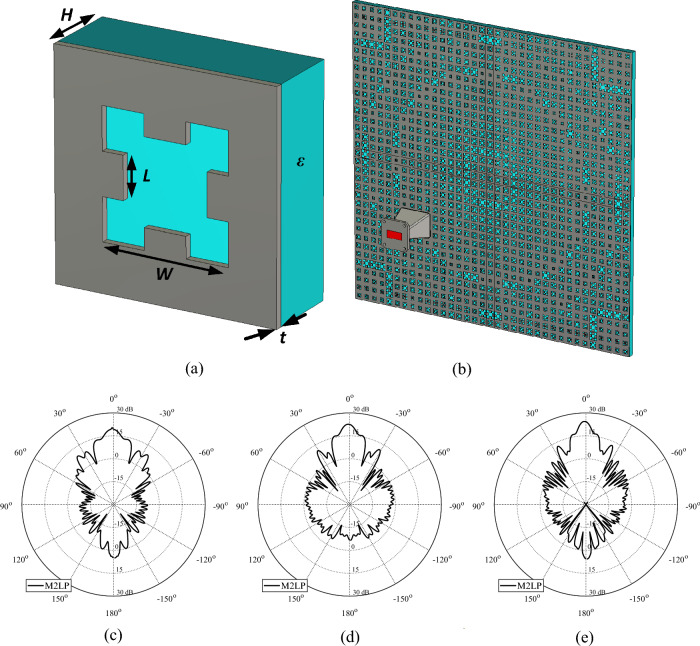
Schematic view of Model 3 unit cell, (**b**) optimized Model 3 TAs obtained using M2LP: EM-simulated realized gain of designs at (**c**) 10 GHz, (**d**) 12 GHz, (**e**) 14 GHz.

As it mentioned before the first TA unit example was selected to illustrate the presented concepts and workflows using simple test case, so that the challenges pertinent to the examples themselves do not make demonstration of the concepts unclear. At this stage in order to clearly demonstrate applicability of the proposed methodology to different types of unit elements two additional example is presented, whereas these examples provide more challenging design cases that can only be solved via EM simulations and AI techniques (Figs. 12 and 13). The first design (Fig. 12a) is based on a recently reported work that aimed to create a high performance TA design with meta-material design and FW-EM simulation approach^66^. This will be referred to as Model 2 throughout the rest of the paper. Figure 12a shows the architecture of Model 2. To achieve high directivity, 10 layers of identical unit cells (Fig. 12b) are taken as the unit element for surrogate modelling purposes. Rogers’s 4350 substrate of the height of 0.42 mm is employed to implement the design. The variables of the Model 2 are *R* (the diameter of the square ring with the range of 1.4–2.4 mm) and *w* (the width of the ring with the range of 0.2–0.8 mm). Each data sample is a vector of the size 1 × 41 representing the evaluation of the unit cell within the frequency band of 8–14 GHz, which is uniformly distributed with step size of 0.1 GHz. The sampling step size of 0.05 mm is applied for each geometry parameters, leading to the total of 373 samples generated for training, and 100 randomly generated sample points for the hold-out of the M2LP surrogate. MAE values for Model 2 are found as 0.014 and 4.8 for |S_21_| and ∠S_21_ respectively. With deployment of the M2LP unit cell surrogate model of Model 2 TA alongside of BO based optimization with a similar cost in Eq. (3) but without third term of weighting the TA design in Fig. 12c is obtained. The simulated radiation characteristics of the model for 8 GHz, 10 GHz, and 14 GHz are found as 20.7 [dBi], 22.6 [dBi], 23.1 [dBi] respectively and depicted in Fig. 12d–f. Here, it is worth mentioning that as opposed to ^66^, the unit elements in each layer are taken as identical elements, which limits the performance of the TA design at a certain level. However, for a unit element consisting of ten layers, individualizing geometry parameters for each of them makes the modeling problem extremely challenging, addressing of which is outside the scope of this paper. Thus, for illustration of the proposed surrogate modeling approach the unit elements are taken as identical at all layers. Yet, even with identical unit cell the Model 2 TA achieves high performance, which can be furthered improved. One of the objectives of the future work is to consider a decomposition-based method for computationally efficient surrogate modeling of multi-layer TA design with distinctive unit cell in each layer.

The last example, shown in Fig. 13a, is based on a metallic Minkowski fractal design, and a dielectric substrate material ^67^. This model will be referred to as Model 3 throughout the rest of the paper. Figure 13a shows the parameterized architecture of the unit cell. The variables are *H* (the height of the dielectric substrate with the range of 1.0–10.0 mm), *ε* (the dielectric constant of the substrate with the range of 1.2 to 2.7), *w* (the width of the Minkowski fractal with the range of 3.0–14.0 mm), *L* (the length of the Minkowski fractal inner part equal to *w* × α where the range of α is 0.1–0.9), and *t* (the thickness of the metallic surface that varies between the range of 0.035 to 0.5 mm). Each data sample is a vector of the size 1 × 41 representing the evaluation of the unit cell within the frequency band of 10–14 GHz, which is uniformly distributed with step size of 0.1 GHz. The Latin Hypercube Sampling technique is applied to generate the dataset, leading to the total of 800 samples used for training, and 100 randomly generated sample points for the hold-out of the M2LP surrogate. MAE values for Model 3 are found as 0.021 and 5.6 for |S_21_| and ∠S_21_, respectively. Similarly to the first and second designs, the same approach is employed for this unit element to create the TA design shown in Fig. 13b. The simulated radiation characteristics of the TA obtained for 10 GHz, 12 GHz, and 14 GHz are shown in Fig. 13c and e, respectively. The corresponding gain values are 19.4 [dBi], 21.6 [dBi], 23.4 [dBi], respectively. It should be reiterated that—as opposed to the first and second unit elements—Model 3 is considerably more involved of higher dimensionality and complexity. Consequently, it cannot be handled using traditional analytical or FW-EM approach modeling. On the other hand, behavioral modeling as presented in this work, offers a practical way of handling the structure and conducting the complete TA design. At the same time, this example corroborated applicability of the proposed methodology to handle more complex cases, including unit elements described by a larger number of geometry parameters. One of the objectives of the future work is to create a surrogate model within higher dimensionality parameter space not only to handle more complex geometrical designs but also to allow utilization of different unit element architectures (e.g., 1: Minkowski fractal, 2: simple square, 3: elliptical shapes, 4: rings, etc.) within the same array. In other words, rather than using a single unit cell architecture within the TA, a variety of structures selected from a pre-defined unit cell library would be employed, all described by the (compound) surrogate model. An additional input variable corresponding to the type of the unit cell structure would be used to make a selection of the unit cell architecture. This would allow to replace a specific unit element by its more suitable counterparts in case if certain transmission phase (or other) requirements cannot be met.

## Conclusion

This paper introduced a novel technique for rapid optimization of transmitarray (TA) designs using low-cost and accurate surrogate models. The proposed methodology capitalizes on a neural-network-based metamodel of flexible architecture, developed to represent the magnitude and phase of the transmission response of the unit elements being the building blocks of the TA. The particular structure of the model, including the arrangement of layers, the number of neurons, hyper-parameters, etc., is automatically adjusted through Bayesian optimization. The model is shown to exhibit competitive performance with transmission phase error not exceeding five degrees, while being established using less than three hundred training data samples. Meanwhile, the predictive power of the benchmark technique, including the most popular SVRM surrogate, is three to six time worse. At the same time, the model covers a very broad frequency range from 1 to 40 GHz, which makes it suitable for the design of TAs operating in a variety of frequency bands within this spectrum.

For illustration purposes, three transmitarray designs are generated using the same model, and demonstrated to exhibit satisfactory performance. For the sake of comparison, the arrays obtained using the SVRM model shown inferior performance both in terms of the radiation gain and the side lobe levels. A selected design is manufactured and experimentally validated to corroborate design utility of the presented technique. The approach proposed in this work may be considered a viable alternative to existing techniques when it comes to cost-efficient and reliable design of large transmitarrays. Here it worth mentioning that, additional parameters such as diffraction or coupling effects of neighbouring unit elements are not taken into consideration in this work. If these parameters were also taken into the study the overall performance of the model might be enhanced. Nonetheless, authors aims to add these effects to their modeling approach in their future work for modeling of TAs/lenses/Reflectarray designs.

## Data Availability

The datasets generated during and/or analysed during the current study are available from the corresponding author on reasonable request.

## References

[1] Abdelrahman AH, Yang F, Elsherbeni AZ, Nayeri P (2017). Analysis and design of transmitarray antennas. Synth. Lect. Antennas.

[2] Aghanejad I, Abiri H, Yahaghi A (2012). Design of high gain lens antenna by gradient-index metamaterials using transformation optics. IEEE Trans. Antennas Propag..

[3] Li Y, Luk KM (2014). Low-cost high-gain and broadband substrate- integrated-waveguide-fed patch antenna array for 60-GHz band. IEEE Trans. Antennas Propag..

[4] Abdelrahman AH, Elsherbeni AZ, Yang F (2014). High-gain and broadband transmitarray antenna using triple-layer spiral dipole elements. IEEE Antennas Wirel. Propag. Lett..

[5] Mahouti P, Belen MA, Çalık N, Koziel S (2022). Computationally efficient surrogate-assisted design of pyramidal-shaped 3D reflectarray antennas. IEEE Trans. Antennas Propag..

[6] Aziz A, Yang F, Xu S, Li M, Chen HT (2019). A high-gain dual-band and dual-polarized transmitarray using novel loop elements. IEEE Antennas Wirel. Propag. Lett..

[7] Datthanasombat, S., Prata, A., Arnaro, L. R., Harrell, J. A., Spitz, S., & Perret, J. “Layered lens antenna,” *Proc.* IEEE Antennas and Propagation Society International Symposium. Digest., 777–780 (Boston, MA, USA, 2001)

[8] Rudge AW, Adatia NA (1978). Offset-parabolic-reflector antennas: A review. Proc. IEEE.

[9] Ryan CGM, Chaharmir MR, Shaker J, Bray JR, Antar YMM, Ittipiboon A (2010). A wideband transmitarray using dual-resonant double square rings. IEEE Trans. Antennas Propag..

[10] Huang J, Encinar JA (2008). Reflectarray Antennas.

[11] Yu A, Yang F, Elsherbeni AZ, Huang J, Kim Y (2012). An offset-fed X-band reflectarray antenna using a modified element rotation technique. IEEE Trans. Antennas Propag..

[12] Abdelrahman AH, Elsherbeni AZ, Yang F (2014). Transmission phase limit of multilayer frequency selective surfaces for transmitarray designs. IEEE Trans. Antennas Propag..

[13] Jingwei X, Whiting EB, Campbell SD, Werner PL, Werner DH, Bossard JA, Barrett JP, Withrow JW, Weigner JD (2022). Multiobjective optimization of bespoke gradient-index lenses: A powerful tool for overcoming the limitations of transformation optics. Phys. Rev. Appl..

[14] Whiting EB, Mackertich-Sengerdy G, Campbell SD, Soltani S, Haack MP, Barrett JP, Withrow JW, Bossard JA, Werner DH (2022). Adjoint sensitivity optimization of three-dimensional directivity-enhancing, size-reducing GRIN lenses. IEEE Antennas Wirel. Propag. Lett..

[15] Salucci M, Tenuti L, Oliveri G, Massa A (2018). Efficient prediction of the EM response of reflectarray antenna elements by an advanced statistical larning method. IEEE Trans. Antennas Propag..

[16] Pozar D, Metzler T (1993). Analysis of a reflectarray antenna using microstrip patches of variable size. Electron. Lett..

[17] Berry D, Malech R, Kennedy W (1963). The reflectarray antenna. IEEE Trans. Antennas Propag..

[18] Oliveri G, Gelmini A, Polo A, Anselmi N, Massa A (2020). System-by-design multiscale synthesis of task-oriented reflectarrays. IEEE Trans. Antennas Propag..

[19] Prado DR, López-Fernández JA, Arrebola M, Goussetis G (2019). Support vector regression to accelerate design and crosspolar optimization of shaped-beam reflectarray antennas for space applications. IEEE Trans. Antennas Propag..

[20] Prado DR, Lopez-Fernández JA, Barquero G, Arrebola M, Las-Heras F (2018). Fast and accurate modeling of dual-polarized reflectarray unit cells using support vector machines. IEEE Trans. Antennas Propag..

[21] Prado DR, Lopez-Fernández JA, Barquero G, Arrebola M (2020). Systematic study of the influence of the angle of incidence discretization in reflectarray analysis to improve support vector regression surrogate models. Electronics.

[22] Prado DR, Lopez-Fernández JA, Barquero G, Arrebola M (2021). On the use of the angle of incidence in support vector regression surrogate models for practical reflectarray design. IEEE Trans. Antennas Propag..

[23] Zhou M, Sørensen SB, Kim OS, Jørgensen E, Meincke P, Breinbjerg O, Toso G (2014). The generalized direct optimization technique for printed reflectarrays. IEEE Trans. Antennas Propag..

[24] Shi L, Zhang Q, Zhang S, Liu G, Yi C (2021). Accurate characterization of graphene reconfigurable reflectarray antenna element by SVR. IEEE J. Multiscale Multiphys. Comp. Tech..

[25] Gosal G, Almajali E, McNamara D, Yagoub M (2016). Transmitarray antenna design using forward and inverse neural network modeling. IEEE Antennas Wirel. Propag. Lett..

[26] Noh J, Nam YH, So S, Lee C, Lee SG, Kim Y, Kim TH, Lee JH, Rho J (2021). Design of a transmissive metasurface antenna using deep neural networks. Opt. Mater. Express.

[27] Yuan L, Wang L, Yang XS, Huang H, Wang BZ (2021). An efficient artificial neural network model for inverse design of metasurfaces. IEEE Antennas Wirel. Propag. Lett..

[28] Abdelrahman, A. H., Nayeri, P., Elsherbeni, A. Z., & Yang, F., “Analysis and design of wideband transmitarray antennas with different unit-cell phase ranges, *Proc.**IEEE Int. Symp. Antennas Propagation*, 1266–1267 (Memphis, TN, USA, 2014).

[29] Balanis CA (2012). Advanced Engineering Electromagnetics.

[30] Zhang S, Njoku CC, Whittow WG, Vardaxoglou JC (2015). Novel 3D printed synthetic dielectric substrates. Microw. Opt. Technol. Lett..

[31] Meireles MRG, Almeida PEM, Simoes MG (2003). A comprehensive review for industrial applicability of artificial neural networks. IEEE Trans. Ind. Electron..

[32] Hopfield JJ (1988). Artificial neural networks. IEEE Circuits Syst. Mag..

[33] Creech GL, Paul BJ, Lesniak CD, Jenkins TJ, Calcatera MC (1997). Artificial neural networks for fast and accurate EM-CAD of microwave circuits. IEEE Trans. Microw. Theory Technol..

[34] Rakitianskaia, A., & Engelbrecht, A., Measuring saturation in neural networks, *Proc*. *2015 IEEE symposium series on computational intelligence*, 1423-1430 (Cape Town, South Africa, 2015).

[35] Glorot, X., Bordes, A., & Bengio, Y., “Deep sparse rectifier neural networks,” *Proc. of the Fourteenth International Conference on Artificial Intelligence and Statistics*, 315–323, (2011).

[36] Javid, A. M., Das, S., Skoglund, M., & Chatterjee, S., A ReLU dense layer to improve the performance of neural networks, *Proc. ICASSP 2021-2021 IEEE International Conference on Acoustics, Speech and Signal Processing*, 2810–2814 (2021).

[37] Zhang, X., Zou, Y., & Shi, W., Dilated convolution neural network with LeakyReLU for environmental sound classification, *Proc.2017 22nd international conference on digital signal processing (DSP)*, 1–5, (2017).

[38] Calik N, Belen MA, Mahouti P (2020). Deep learning base modified MLP model for precise scattering parameter prediction of capacitive feed antenna. Int. J. Numer. Model. Electron. Netw. Devices Fields.

[39] Shahriari B, Swersky K, Wang Z, Adams RP, de Freitas N (2016). Taking the human out of the loop: A review of bayesian optimization. Proc. IEEE.

[40] Zhou M (2014). The generalized direct optimization technique for printed reflectarrays. IEEE Trans. Antennas Propag..

[41] Zhang Y, Xu X (2020). Solubility predictions through LSBoost for supercritical carbon dioxide in ionic liquids. New J. Chem..

[42] Al-Mahasneh, A. J., Anavatti, S. G., & Garratt, M. A., Review of applications of generalized regression neural networks in identification and control of dynamic systems, *arXiv preprint*, arXiv:1805.11236, (2018).

[43] Chang CC, Lin CJ (2011). LIBSVM: A library for support vector machines. ACM Trans. Intell. Syst. Technol..

[44] Nguyen T, Hanzhi Ma BS, Li EP, Chen X, Cangellaris AC, Schutt-Ainé J (2021). Comparative study of surrogate modeling methods for signal integrity and microwave circuit applications. IEEE Trans. Compon. Packag. Manuf. Technol..

[45] Calik N, Güneş F, Koziel S, Dabrowska AP, Belen MA, Mahouti P (2023). Deep-learning-based precise characterization of microwave transistors using fully-automated regression surrogates. Sci. Rep..

[46] Nayeri P, Yang F, Elsherbeni AZ (2010). Broadband reflectarray antennas using double-layer subwavelength patch elements. IEEE Antennas Wirel. Propag. Lett..

[47] Nayeri P, Yang F, Elsherbeni AZ (2011). Bandwidth improvement of reflectarray antennas using closely spaced elements. Prog. Electromagn. Res. C.

[48] Mao Y, Xu S, Yang F, Elsherbeni AZ (2015). A novel phase synthesis approach for wideband reflectarray design. IEEE Trans. Antennas Propag..

[49] Belen A, Mahouti P, Güneş F, Tari Ö (2021). Gain enhancement of a traditional horn antenna using 3d printed square-shaped multi-layer dielectric lens for x-band applications. App. Comp. Electromag. Soc. J..

[50] Belen A, Güneş F, Mahouti P, Palandöken M (2020). A novel design of high performance multilayered cylindrical dielectric lens antenna using 3D printing technology. Int. J. RF Microw. Comput. Aid. Eng..

[51] Li WT, Tang HS, Cui C, Hei YQ, Shi XW (2022). Efficient online data-driven enhanced-XGboost method for antenna optimization. IEEE Trans. Antennas Propag..

[52] Sharma A (2022). Antenna array pattern synthesis using metaheuristic algorithms: a review. IETE Tech. Rev..

[53] Yang G, Zeng H, Xu Z (2021). Adaptive gradient search algorithm for displaced subarrays with large element spacing. IEEE Antennas Wirel. Propag. Lett..

[54] Zhou J, Yang Z, Si Y, Kang L, Li H, Wang M, Zhang Z (2021). A trust-region parallel bayesian optimization method for simulation-driven antenna design. IEEE Trans. Antennas Propag..

[55] RoboxDual by CEL—a dual material 3D printer, https://cel-uk.com/shop/roboxdual-by-cel-a-dual-material-3d-printer/, available on (16.08.2022).

[56] PLA 1.75mm 3D printing filament, https://cel-uk.com/shop/pla/, available on (16.08.2022).

[57] Wei F, Hao J-W, Xu L, Shi X (2021). A circularly polarized 3-d printed dielectric transmitarray antenna at millimeter-wave band. IEEE Antennas Wirel. Propag. Lett..

[58] Liu X, Peng L, Liu YF, Yu WS, Zhao QX, Jiang X, Li SM, Ruan C (2021). Ultrabroadband all-dielectric transmitarray designing based on genetic algorithm optimization and 3-d print technology. IEEE Trans. Antennas Propag..

[59] Massaccesi, A. & Pirinoli, P. Space-fed antenna based on dielectric-only transmitarray, *Proc. 2022 16th European Conference on Antennas Propagation*, 1–4 (2022).

[60] Tian C, Lu Y-Q, Zhao G, Jiao Y-C, Guo L-X (2022). Double-layer transmitarray antenna using specially designed substrate. IEEE Antennas Wirel. Propag. Lett..

[61] Yang J, Chen ST, Chen M, Ke JC, Chen MZ, Zhang C, Yang R, Li X, Cheng Q, Cu TJ (2021). Folded transmitarray antenna with circular polarization based on metasurface. IEEE Antennas Wirel. Propag. Lett..

[62] Yu L, Li X, Zhu H, Qi Z (2020). A design of oam metal-only transmitarray antenna using high-transmission slot-type jerusalem elements. Appl. Comput. Electromagn. Soc. J..

[63] Tian C, Jiao Y-C, Zhao G, Wang H (2017). A wideband transmitarray using triple-layer elements combined with cross slots and double square rings. IEEE Antennas Wirel. Propag. Lett..

[64] Zheng B, Fan Y, Cheng YJ (2023). Wideband high-efficiency circularly polarized transmitarray with linearly-polarized feed. IEEE Antennas Wirel. Propag. Lett..

[65] Yang S, Yan Z, Liu P, Li X (2022). A linearly-polarized-feed dual-circularly polarized dual-beam transmitarray with independent beam control. IEEE Antennas Wirel. Propag. Lett..

[66] Papathanasopoulos A, Rahmat-Samii Y, Garcia NC, Chisum JD (2020). A novel collapsible flat-layered metamaterial gradient-refractive-index lens antenna. IEEE Trans. Antennas Propag..

[67] Cao Y, Zhang J, Tian C, Liu Y, Zhang J, Zhang W (2022). Design of a frequency selective surface-backed microstrip reflectarray antenna using Minkowski ring elements. Int. J. RF Microw. Comp. Aid. Eng..

